# Gender differences in managers’ attitudes towards employees with depression: a cross-sectional study in Sweden

**DOI:** 10.1186/s12889-020-09848-2

**Published:** 2020-11-19

**Authors:** Ilaria Mangerini, Monica Bertilsson, Angelique de Rijk, Gunnel Hensing

**Affiliations:** 1 Department of Social Medicine, Care and Public Health Research Institute (CAPHRI), Faculty of Health, Medicine and Life Sciences, Maastricht University, Maastricht, The Netherlands; 2 School of Public Health and Community Medicine, Institute of Medicine, The Sahlgrenska Academy, University of Gothenburg, Gothenburg, Sweden

**Keywords:** Managers, Stigma, Negative attitudes, Employee, Depression, Gender, Mental health

## Abstract

**Background:**

Depression is prevalent among employees and a major reason for sickness absence. First-line managers’ attitudes towards employees with depression might influence return to work and the scant literature indicates gender differences in attitudes. The objective of this study was to investigate gender differences in managers’ attitudes to employees with depression.

**Methods:**

A cross-sectional study was conducted among 4737 Swedish managers in 2017 (response rate 71%, *n* = 3358). Attitudes towards depression were measured with the instrument “Managerial stigma towards employees with depression” (12 items). The response patterns of women and men, the level of stigma and the direction of the gender differences were investigated with independent *t* tests and binary logistic regression analyses with covariates.

**Results:**

The likelihood of reporting high negative attitudes (score ≥ 36) was lower among women than men (odds ratio, 1.64; 95% confidence interval, 1.28–2.10) after adjusting for age, level of education, work sector, distribution of women and men among the staff, current workplace experience in management, lifetime experience in management, managerial position and presence of staff members at the current workplace who had depression and/or anxiety disorders.

**Conclusions:**

Based on these findings, a gender-sensitive approach is suggested for future interventions to improve managers’ attitudes towards employees with depression and other mental disorders.

**Supplementary Information:**

The online version contains supplementary material available at 10.1186/s12889-020-09848-2.

## Background

By 2030, it is estimated that depression will be the second-highest cause of disability-adjusted life years (DALYs) [1] and that around 25% of individuals over 18 years old will develop one or more mental disorder in their life time [2]. Among common mental disorders (CMDs), depression has the highest prevalence [3], and in Sweden, CMD is the most common diagnostic group among newly sick-listed women and the second most common in men (2016) [4]. Depression has been linked to impaired work performance, high levels of absenteeism and early retirement [5]. Furthermore, several studies show that return to work after sickness absence is prolonged for individuals with CMDs [6, 7], and once back at work, many still report decreased work capacity [8]. Recurrence is more common among those sick-listed with CMDs than among other sick-listed groups [9]. Researchers investigating causative factors found that experiencing job strain was related to an increased risk of incident depressive symptoms [10] and sickness absence, and was a drawback in return-to-work processes of employees [11].

First-line managers and employers play a key role in the prevention of (long-term) sickness absence [12], and various European countries have introduced legislation and policy concerning prevention of excessively high psychosocial job demands. The Working Environment Acts of Norway, Sweden and Denmark, the Labour Conditions Law in the Netherlands are examples of legislation that requires employers to support employees with CMDs so that they can remain in work or return to work after sickness absence [12].

### Stigma among managers

Public stigma is the reaction that the general population, e.g. managers, have to people with mental illness, and it can be understood in terms of three components [13]: prejudice, agreement with a belief and/or negative emotional reaction to a group (e.g. anger, fear, dislike); stereotype, a negative belief about a group (e.g. dangerousness, incompetence, character weakness, laziness) endorsed by people who are prejudiced; and discrimination, a behavioural response to prejudice (e.g. avoidance, withholding help or promotion). Prejudice may lead to discrimination, such as withholding employment, lowered supervisor expectations, lack of respect, isolation from co-workers or being passed over for promotion [14] based solely on people’s perceptions of a diagnosis [13]. In this work, we have investigated the gender differences in stigma operationalized as managers’ attitudes towards employees with depression.

With reference to the workplace environment, fear might lead to avoidance. Previous studies showed that perceived discrimination had harmful effects on the psychological well-being of employees [15]. Martin [16] investigated managers’ stigma as negative attitudes towards employees with depression using a novel instrument distinguishing the three types of stigma described earlier for public stigma: prejudicial emotional reaction (affective attitudes); stereotypes or beliefs about depressed employees (cognitive attitudes); and discrimination against employees with mental illness (behavioural attitudes) [16, 17]. She found that managers’ attitudes were clearly important for effectively managing depression within the workplace, and that managers who were more educated, worked in the public sector and/or who were female were less likely to report negative attitudes than those who were less educated, worked in the private sector and were male [16].

### Gender and manager attitudes towards depression among employees

The few published studies found that women as managers report a lower degree of negative attitudes than the corresponding group of men. So far, these studies have been performed in Australia and Germany [16, 18]. We lack studies from Scandinavia, which is considered to be among the most developed regions in gender equality and where the male breadwinner model is less supported [19, 20]. The increase in the number of women participating in paid work and of female managers occurred earlier in this part of the world (e.g. in Sweden there was an increase of 16% between 2002 and 2013 [21]). It is important to study gender differences in attitudes towards depression due to the global increase in the number of female managers in recent years [22, 23].

The role expectations often associated with gender and leadership are another reason for this study. To be perceived as credible and legitimate entrepreneurial leaders, female managers are expected to meet social role expectations of being a woman (being interpersonally sensitive and caring), while also meeting dominant masculine constructions of leadership and entrepreneurship (being aggressive and dominant) [24–27]. Male gender-typed positions, which include top management and executive positions, are believed to necessitate characteristics that coincide with stereotypic conceptions of men but not with stereotypic conceptions of women [28].

Because of the increase in female representation in management, the gendered role expectations in leadership and earlier findings suggesting a gender difference in attitudes [16, 22, 23], it is important to take a gender perspective when studying managers’ attitudes towards depression in their employees.

We take the perspective of gender, referring to the broad division of human experiences into male and female, as a constant point of reference. This is in contrast to the dichotomy of sex differences, because this is inadequate to represent the reality of human life [29] and diversity within the gender categories [30]. The belief that due to sex, biological differences are reflected in profound psychological characteristics and behaviour differences, has been tested in a large body of research, and decisively refuted [30]. Gender can instead be understood as a structure of social relations, which under different historical circumstances, takes vastly different forms [31]. The concept of structure refers to large-scale patterns such as the contrast between masculinity and femininity and the gender division of labour at work and at home, which can be found across institutions as families, companies, governments and neighbourhoods. Gender structures are cultural points of reference for daily life, but they also operate as emotional and material constraints embedded in person-to-person relationships, and in the built environment [30] when observed in a workplace or organization, and can even be defined as a gender regime that might change over time, e.g. due to changes in gender composition in the workplace [31].

### Aim and research questions

The aim of this study was to investigate negative attitudes towards employees with depression with a specific focus on managers’ gender. Two research questions were formulated:

1. Do the response patterns for the measure “negative attitudes towards employees with depression” [16] differ between men and women? And how do possible gender differences vary in relation to age and staff composition by gender?
2. What is the association between gender and managers’ negative attitudes to depression?

## Methods

### Design

This cross-sectional study is part of “Managers’ Perspective–The Missing Piece”, a sub-project of the New Ways research programme on mental health at work.

### Procedure of data collection

The Laboratory of Opinion Research (LORE) at the University of Gothenburg distributed an online questionnaire to two samples of managers: the Citizen Panel sample and the HELIX sample. The LORE conducts data collection through web-based surveys and their Citizen Panel consists of about 60,000 self-recruited individuals throughout Sweden [32]. The partnership organization HELIX Competence Centre focuses on research on sustainable working life and is situated in and around Linköping, Southeast of Sweden [33].

In June 2017, the LORE used two questions in Citizen Panel n° 26 to screen and identify managers. From this screened sub-panel, 5000 managers were randomly sampled to this study. The HELIX Competence Centre invited their 22 partnership organizations to participate in this study. Eight of the 22 organizations accepted participation. These 8 employers supplied 556 e-mail addresses to managers in their organizations. The LORE sent the online questionnaire to these addresses.

**Fig. 1:**
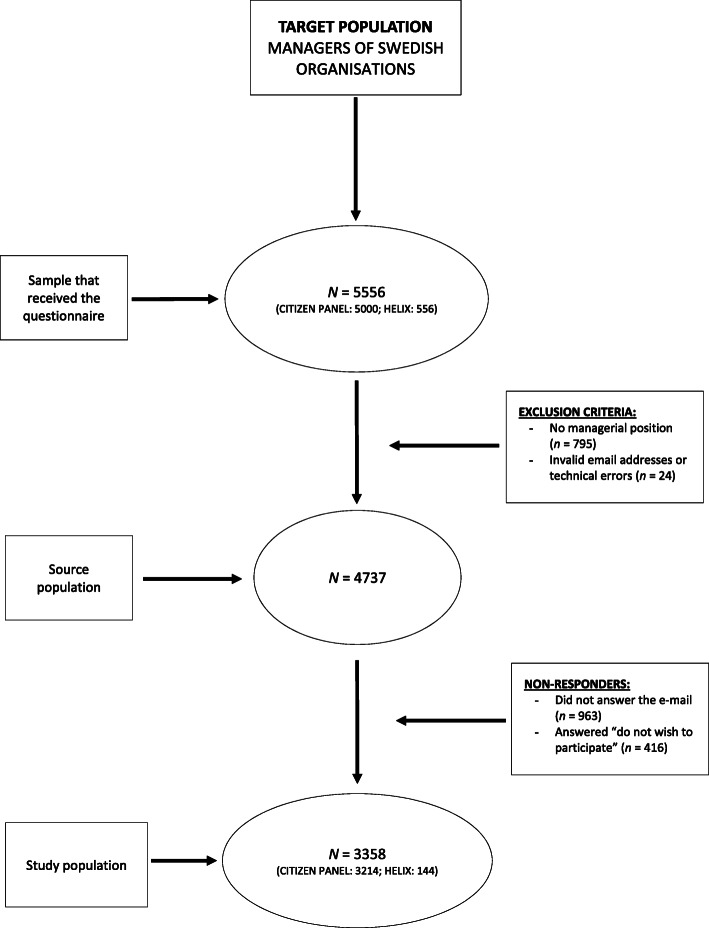
Flowchart of inclusion procedures of the New Ways project “Managers’ perspective – the missing piece”, Sweden, 2018

Of the source population fulfilling the inclusion criteria (being a manager *n* = 4737), 3358 participated, with a response rate of 71% (Fig. 1).

### Measurements

#### Exposure variable

Gender, as well as all the other variables, was self-reported. From this sample, managers who answered non-binary to the gender question were excluded from the analysis due to the limited size (*n* = 3).

#### Outcome variable

Negative attitudes towards employees with depression was measured with “Managerial stigma towards employee depression” (MSED) instrument [16] shown in Table 1, a newly developed 12-item scale measuring managers’ affective, cognitive and behavioural stigma against employees with depression, addressing the three types of stigma described by Corrigan et al. [13].

**Table 1:** Martin’s questionnaire: “Managerial stigma towards employee depression” [16]

Type of stigma	Item no.	Statement
Affective attitudes	1 (R)	I feel comfortable when I have to deal with staff members who have depression
	2	It feels frightening to deal with staff members who have depression
	3	It is stressful to work with staff members who have depression
	4	I feel uncomfortable when I work next to a staff member who is depressed
Cognitive attitudes	5	If a staff member suffers from depression, the reason rests mainly on herself/himself
	6	Staff members with depression are a burden for the workplace
	7	Staff members taking antidepressant medication should not be working
	8	Staff members with depression can get over their depression if they just want to
Behavioural attitudes	9	I would avoid talking to a staff member who has depression so that I don’t have to deal with the person’s problems
	10	I would not hire someone who I knew had been depressed
	11 (R)	I would make temporary changes in the job to help a depressed staff member to recover
	12	I would like to get rid of a staff member who has depression

Items were culturally translated for Sweden. R, reverse phrased, recoded

The MSED instrument is currently the only depression stigma instrument addressing managers specifically [16, 17]. Co-authors MB and GH of this paper and a third researcher culturally translated the items from English to Swedish with support from two Swedish managers and one human resources specialist. The entire questionnaire was pilot tested among 9 Swedish managers. The final Swedish version was back-translated to English by an official translating firm and considered conceptually and culturally equivalent to the original instrument by the researcher who initially developed the instrument [16]. The MSED instrument encompasses 12 attitude statements with a 6-point Likert scale (1 = strongly disagree and 6 = strongly agree). The two reverse-phrased items were recoded.

The MSED instrument was previously used in a sample of 469 Australian managers and resulted in good psychometric adequacy and good internal reliability [17]. In our study, Cronbach’s alpha coefficient was 0.800, which suggests good internal consistency and reliability [34]. Cronbach’s alpha was checked with deletion of each item, and this confirmed that all items fitted the scale.

To perform the analyses, MSED scores were dichotomized at the 3rd quartile into reporting high negative attitudes (sum scores, ≥36) versus low negative attitudes (sum scores, 12–35). The minimum score was 12 and the maximum score was 72.

#### Covariates

Covariates were chosen theoretically to control for the effects of individual and social factors on the relationship between gender and stigma as suggested by Angermeyer et al. [18].

Age was measured as follows: younger than 20 years; 20–29 years; 30–39 years; 40–49 years; 50–59 years; 60–65 years and older than 65 years. Age was recoded to combine the categories with a small number (there were no individuals younger than 20 years and only one older than 65 years) as follows: 20–29 years; 30–39 years; 40–49 years; 50–59 years and 60 years and older. In the logistic analyses, age was dichotomized into younger than 50 years and 50 years and older.

Level of education was measured as follows: compulsory school; upper secondary school or equivalent; degree from college/university (minimum 3 years) and other post-secondary education. Level of education was recoded to combine the compulsory school category with the following category because of the small number as follows: upper secondary school or less; degree from college/university (minimum 3 years) and other post-secondary education.

Work sector was measured as follows: governmental; municipal; county council/regional; private and non-profit organization/foundation. All the answer options were retained in the logistic analyses.

Managerial position was measured as follows: senior manager (such as administration manager, managing director); middle management (manager of managers); middle management/first-line manager; group leader/supervisor; expert/operations manager (such as personnel manager, finance manager). All the answer options were retained in the logistic analyses.

Current workplace experience in management was measured as follows: 0–1 year, 2–3 years, 4–5 years and more than 5 years. In the logistic analyses, the variable was dichotomized into 5 years or less and more than 5 years.

Lifetime experience in management was measured as follows: 0–2 year, 3–5 years, 6–10 years and more than 10 years. In the logistic analyses, the variable was dichotomized into 10 years or less and more than 10 years.

Distribution of women and men among the staff was measured as follows: most are women, there are about as many women as men and most are men at the current workplace. All the answer options were retained in the logistic analyses.

Presence of staff members at the current workplace who have had depression and/or anxiety disorders was measured as follows: several staff members, one staff member, no staff member and don’t know. In the logistic analyses, the variable was recoded into three categories: one or more staff members, no staff member and don’t know.

### Analysis

Participants who did not answer the specific study questions or did not provide information related to the covariates considered in this study were not included in the analysis. The final study population was composed of 2663 participants (56% of the source population). (Fig. 1) To answer the first research question, we computed the % agreement per answering category per item of the MSED instrument, and next, we performed chi-squared tests for the percentage of high negative attitude scores (see ‘Measurements’ on how this variable was dichotomized) across gender, and for gender combined with individual and organizational characteristics. To answer the second research question, binary logistic regression analyses on negative attitudes were performed with the enter method for gender and the backward stepwise method with conditional removal criteria (*p* < 0.10) for different blocks of possible covariates.

The data analyses were performed with SPSS version 24 (IBM).

## Results

Table 2 presents the distribution of women and men for individual and organizational characteristics and the analysis of statistical significance. There were no statistically significant differences in the age distribution of women and men. The majority of both categories (65%) were 40–59 years old. More women than men held a degree from college or university (minimum 3 years). The first-line manager and the supervisor positions were held by 40.6 and 21.3% of women, respectively; and the same managerial positions were held by 28.2 and 19.5% of men, respectively. More men worked in the private sector and more women worked in the municipal sector. Looking at the gender distribution among the staff, a majority of women and men worked in a workplace where the staff comprised employees of the same gender.

**Table 2:** Distribution of individual and organizational characteristics within the groups of female and male managers (*N* = 2663)

		Women, % (*n* = 901)	Men, % (*n* = 1762)	χ^2^ *p* value
**Individual characteristics**				
Age	20–29 years	0.9	1.4	< 0.327
	30–39 years	16.6	14.4	
	40–49 years	31.2	33.7	
	50–59 years	37.0	36.0	
	60 years and older	14.3	14.5	
Level of education	Upper secondary school or lower	7.9	19.0	< 0.000
	Degree from college/university^a^	75.0	58.6	
	Other post-secondary education	17.1	22.4	
Managerial position	Senior manager^b^	13.9	27.5	< 0.000
	Middle management^c^	15.0	17.7	
	Middle management/first-line manager	40.6	28.2	
	Group leader/supervisor	21.3	19.5	
	Expert/operations manager^d^	9.2	7.1	
Current workplace experience in management	0–1 year	23.5	17.0	< 0.000
	2–3 years	28.0	23.7	
	4–5 years	12.7	12.1	
	More than 5 years	35.8	47.2	
Lifetime experience in management	0–2 years	15.1	8.4	< 0.000
	3–5 years	20.8	16.4	
	6–10 years	20.5	20.9	
	More than 10 years	43.6	54.3	
**Organizational characteristics**				
Work sector	Governmental	15	12.8	< 0.000
	Municipal	29.9	10.9	
	County council/regional	6.2	4.1	
	Private	39.8	67.0	
	Non-profit organization/foundation	9.1	5.3	
Distribution of women and men among the staff	Most are women	56.5	24.3	< 0.000
	There are about as many women as men	26.2	28.3	
	Most are men	17.3	47.4	
Presence of staff members at current workplace who have had depression and/or anxiety disorders	Yes, several staff members	39.8	27.2	< 0.000
	Yes, one staff member	33.2	32.5	
	No, no staff member	21.5	32.1	
	Don’t know	5.4	8.2	

^a^Minimum 3 years

^b^Such as administration manager, managing director

^c^Manager of managers

^d^Such as personnel manager, finance manager

### Response patterns in women and men

**Fig. 2:**
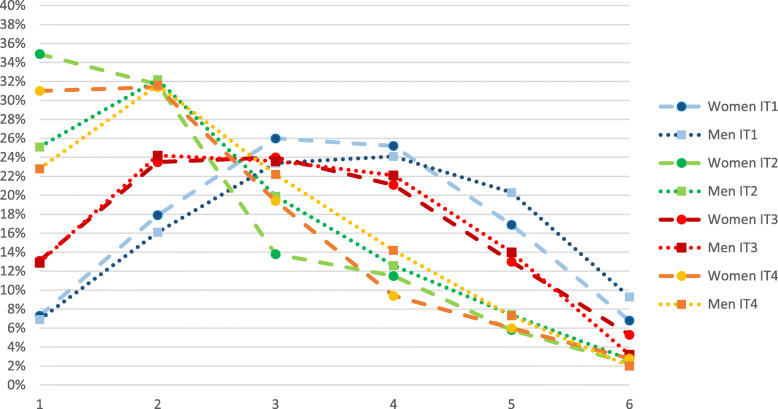
Response patterns for items (IT) 1–4 (affective attitudes) from Martin’s questionnaire, Managerial Stigma Towards Employee Depression

**Fig. 3:**
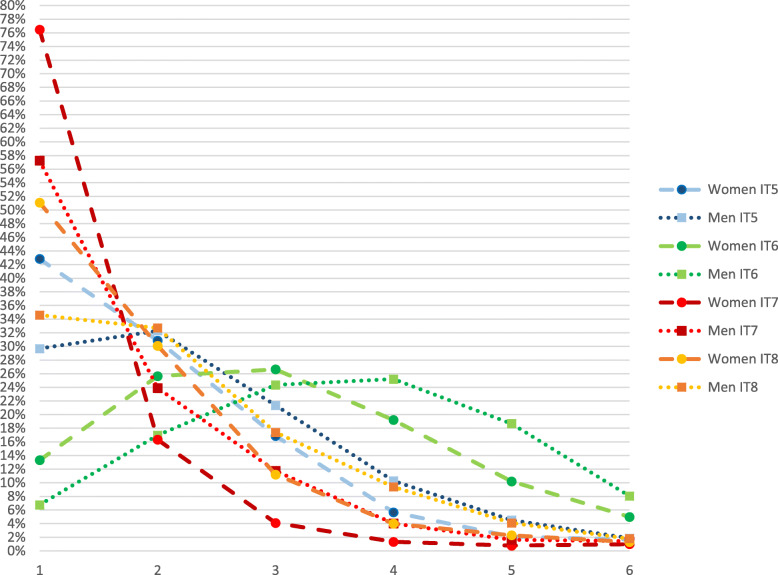
Response patterns for items (IT) 5–8 (cognitive attitudes) from Martin’s questionnaire, Managerial Stigma Towards Employee Depression

**Fig. 4:**
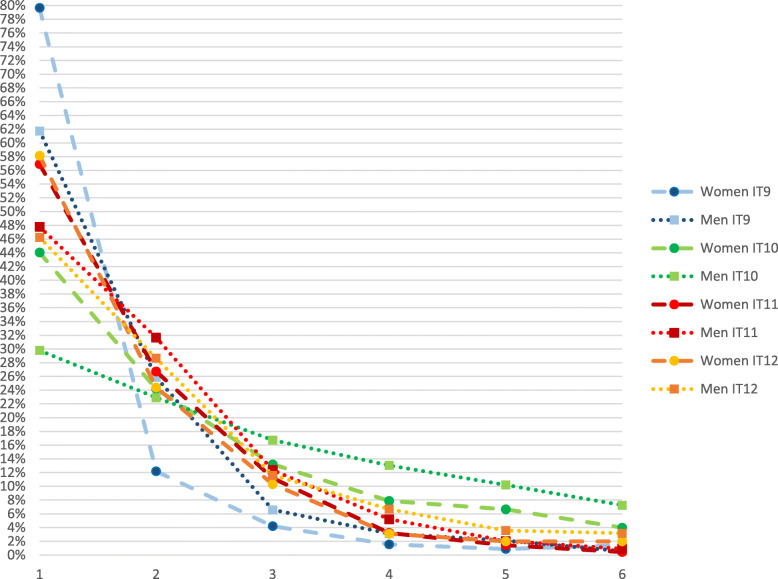
Response patterns for items (IT) 9–12 (behavioural attitudes) from Martin’s questionnaire, Managerial Stigma Towards Employee Depression

The results of the investigation of whether the response patterns were differently distributed across gender either in relation to the item content or to the response pattern are presented in Figs. 2, 3 and 4, for affective, cognitive and behavioural items, respectively. Figures 2, 3 and 4 illustrate the crude response patterns among women and men for the items of the MSED instrument shown in Table 1. The scores in the figures indicate the percentage that agreed with each of the six answering categories (1 = low negative attitude; 6 = high negative attitude) within the group of women and men respectively. For every item, a significantly higher proportion of women than men reported lower negative attitudes, except for item 3 (stressful to work with staff members who have depression) (*p* < 0.185).

### The distribution of negative attitudes in relation to individual and organizational characteristics

Table 3 presents the distribution of negative attitudes in relation to individual and organizational characteristics. In total, 791 women were classified as reporting low negative attitudes (scores 12–35), and 110 women as reporting high negative attitudes (scores 36 and above). The corresponding figures for men were 1333 classified as low negative attitudes and 429 as high negative attitudes. We found that for women, the highest percentage of managers reporting high negative attitudes was among those between 40 and 49 years old, whereas for male managers, the highest percentage was reported in the age range between 30 and 39 years. In contrast, for women, the lowest percentage of managers reporting high negative attitudes was among managers between 30 and 39 years old.

**Table 3:** The distribution of attitudes to depression among Swedish managers in relation to individual and organizational characteristics (*N* = 2663)

	Gender, % (*n* = 1762 men, *n* = 901 women)	Low negative attitudes (score 12–35)	High negative attitudes (score ≥ 36)	χ^2^ *p* value
**Individual characteristics**				
Age				
20–29 years	Women	87.5	12.5	Men < 0.830; Women < 0.631
	Men	76.0	24.0	
30–39 years	Women	91.3	8.7	
	Men	73.2	26.8	
40–49 years	Women	86.1	13.9	
	Men	75.9	24.1	
50–59 years	Women	87.4	12.6	
	Men	76.8	23.2	
60 years and older	Women	88.4	11.6	
	Men	74.5	25.5	
Level of education				
Upper secondary school or lower	Women	74.6	25.4	Men < 0.000; Women < 0.001
	Men	67.5	32.5	
Degree from college/university^a^	Women	89.8	10.2	
	Men	77.3	22.7	
Other post-secondary education	Women	85.1	14.9	
	Men	78.4	21.6	
Managerial position				
Senior manager^b^	Men	67.8	32.2	Men, < 0.000; Women < 0.001
	Women	78.4	21.6	
Middle management^c^	Men	78.8	21.2	
	Women	90.4	9.6	
Middle management/first-line manager	Men	80.5	19.5	
	Women	92.1	7.9	
Group leader/supervisor	Men	77.8	22.2	
	Women	84.9	15.1	
Expert/operations manager^d^	Men	72.8	27.2	
	Women	85.5	14.5	
Current workplace experience in management				
0–1 year	Women	91.0	9.0	Men < 0.002; Women < 0.115
	Men	76.3	23.7	
2–3 years	Women	89.7	10.3	
	Men	81.8	18.2	
4–5 years	Women	86.0	14.0	
	Men	77.0	23.0	
More than 5 years	Women	84.8	15.2	
	Men	72.0	28.0	
Lifetime experience in management				
0–2 years	Women	91.2	8.8	Men < 0.325; Women < 0.099
	Men	79.1	20.9	
3–5 years	Women	91.4	8.6	
	Men	77.5	22.5	
6–10 years	Women	87.0	13.0	
	Men	77.2	22.8	
More than 10 years	Women	85.2	14.8	
	Men	74.0	26.0	
**Organizational characteristics**				
Work sector				
Governmental	Women	87.4	12.6	Men < 0.000; Women < 0.009
	Men	79.1	20.9	
Municipal	Women	92.2	7.8	
	Men	86.5	13.5	
County council/regional	Women	92.9	7.1	
	Men	86.1	13.9	
Private	Women	83.3	16.7	
	Men	71.8	28.2	
Non-profit organization/foundation	Women	90.2	9.8	
	Men	86.0	14.0	
Distribution of women and men among the staff				
Most are women	Women	88.6	11.4	Men < 0.001; Women < 0.277
	Men	79.3	20.7	
There are about as many women as men	Women	88.6	11.4	
	Men	79.5	20.5	
Most are men	Women	84.0	16.0	
	Men	71.5	28.5	
Presence of staff members at current workplace who have had depression and/or anxiety disorders				
Yes, several staff members	Women	90.3	9.7	Men < 0.000; Women < 0.173
	Men	79.8	20.2	
Yes, one staff member	Women	87.0	13.0	
	Men	78.7	21.3	
No, no staff member	Women	84.0	16.0	
	Men	71.2	28.8	
Don’t know	Women	89.8	10.2	
	Men	67.6	32.4	

^a^Minimum 3 years

^b^Such as administration manager, managing director

^c^Manager of managers

^d^Such as personnel manager, finance manager

Chi-squared tests were performed, and significant differences for gender and negative attitudes were found for every characteristic except age. Thus, all individual and organizational characteristics were considered relevant covariates on the basis of the bivariate analyses.

### Association between managers’ negative attitudes to depression and their gender, adjusted for covariates

Table 4 shows that the odds for reporting negative attitudes were higher for men with a crude odds ratio (OR) of 2.31 (95% confidence interval [CI], 1.84–2.91). An appreciable difference can be noted moving from model 1 (OR, 2.15; 95% CI, 1.71–2.71) to model 2 (OR, 1.81; 95% CI, 1.42–2.31). Model 1 shows that compared with managers who have an education level of upper secondary school or lower, managers who have a higher education level show less probability of reporting negative attitudes [16]. In model 2, sector and distribution of women and men among the staff were added and the gender difference maintained. In model 3, which also included current workplace experience in management and managerial position, and model 4, which added the presence of staff members at the current workplace who have had depression and/or anxiety disorders, the gender difference was still present. After adjusting for all covariates, the odds for negative attitudes were higher among men (OR, 1.64; 95% CI, 1.28–2.10). The ORs of the covariates can be found in the Additional file 1: Appendix.

**Table 4:** Crude and adjusted odds ratio (OR) with 95% confidence interval (CI) for low negative attitudes compared with high negative attitudes in Swedish managers (*N* = 2663 of which 1762 were men and 901 were women): results of binary logistic regression analyses, 2018

	Number	Negative attitudes towards depression					
		Unadjusted OR (95% CI)	Model 1: OR (95% CI)^a^	Model 2: OR (95% CI)^b^	Model 3: OR (95% CI)^c^	Model 4: OR (95% CI)^d^	Model 5: OR (95% CI)^e^
Gender							
Women	901	1	1	1	1	1	1
Men	1762	2.31 (1.84–2.91)	2.15 (1.71–2.71)	1.81 (1.42–2.31)	1.70 (1.32–2.17)	1.66 (1.29–2.12)	1.64 (1.28–2.10)

^a^Adjusted for level of education (age removed)

^b^Adjusted for level of education, sector and distribution of women and men among the staff

^c^Adjusted for level of education, sector, distribution of women and men among the staff, current workplace experience in management and managerial position (lifetime experience in management removed)

^d^Adjusted for level of education, sector, distribution of women and men among the staff, current workplace experience in management, managerial position and presence of staff members at current workplace who have had depression and/or anxiety disorders

^e^Adjusted for age, level of education, sector, distribution of women and men among the staff, current workplace experience in management, lifetime experience in management, managerial position and presence of staff members at current workplace who have had depression and/or anxiety disorders

## Discussion

In a group of 2663 Swedish managers, composed of 901 women and 1762 men, we found that the managers’ gender was significantly associated with negative attitudes to depression: women had less negative attitudes than men. We used three types of approaches to explore the relationship: a comparison of item by item from the MSED scale [16]; a comparison of the proportion of women and men who scored above the threshold for negative attitudes; and a multivariate regression with adjustment for covariates that might affect the association. Irrespective of the approach, the associations were in the same direction.

The tendency to actively alienate and discriminate employees with depression (behavioural negative attitudes) was lower for women than for men. We did not find any other studies on the levels of attitudes in managers. However, the general finding corroborates a study from the United States, which found that mental disorders were the second most common basis for charges of discrimination and workplace harassment [35]. In Australia, Martin [16] found that managers who had some previous experience with depression were less likely to report negative behavioural attitudes.

According to social role theory [36], our findings that women reporting low behavioural negative attitudes may be because women are socialized into espousing empathy to a higher extent than men [37]. Based on other studies, these attitudes might lead to minimization of the extent to which their beliefs about individuals with mental illness influence how they behave towards them [37], prompting more favourable reactions [38]. We might speculate that this can be explained by how female and male managers are socialized and educated differently. Moreover, the prevalence of depression in women is generally higher, which might lead to more experience of depression in one’s social network, including the employees at one’s workplace, which might foster tolerance and compassion [25, 39]. Men might have less experience of depression in their daily lives, and therefore might have less knowledge and more misconceptions, which might explain the higher degree of stigma among men [25]. Attitudes that depression is a female ailment might even add to the negative attitudes among men [40].

In this study, more women than men reported that they would make temporary changes to the job to help a depressed staff member to recover. This is corroborated by Van de Voort et al. [41], who found that women were more likely than men to initiate managerial preventive actions in relation to CMDs among employees, such as reviewing employee’s assignments or talking about mental health at the workplace. Ewalds-Kvist et al. [27] found that female respondents among the Swedish population were more empathetic than men towards persons with mental illness in terms of attitudes of open-mindedness and preparedness to integrate persons with mental illness into the community, but they were also more fearful and avoidant in this regard than men. So, even if the will is present, it might not be enough to turn attitudes into actions. According to Telwatte et al. [42], regular contact with persons with disabilities, knowledge of disability legislation, and positive attitudes (but not discomfort) were associated with both seeing work accommodation requests as more reasonable and having greater willingness to grant accommodation. Another issue highlighted was the lower perceived legitimacy of psychological disabilities, which meant that accommodation in relation to psychological disabilities was granted much less commonly than for physical disabilities [42].

Our study suggests that men in male-dominated workplaces might risk exposure to stigmatizing processes that lead to unmet needs for mental health care, which has been demonstrated in another study [43]. Other studies have found that work accommodation is less approved for employees with CMDs compared with other disorders [42, 44], therefore such studies should include the managers’ gender in the future to better understand the field.

The reasoning suggested in the introduction that the gender equality mentality in Sweden influenced managers towards equal attitudes to depression was not sustained in the results, because female managers were 61 to 43% less likely to report negative attitudes across all models tested (Table 4). Moreover, this gender difference remained stable after including other variables. For men, Vogel et al. [40] concluded that, due to conformity to dominant masculine norms, behaviours associated with vulnerability and weakness (such as mental illnesses) were often viewed in a negative light. Women are subject to much stronger expectations than men that they will behave altruistically [45], and another study found that women are well aware of these gender stereotype-based behavioural prescriptions, and their concern over encountering backlash effects from violating these stereotypes helps explain, in part, a range of behaviours that systematically vary by gender [46]. The relationship with educational level and experience with employees with depression/anxiety disorders was in line with expectations [16]. Theorell et al. found a socioeconomic gradient in a study on non-listening leadership. This leadership style might be related to attitudes to depression. Future studies are needed for a better understanding of leadership styles and attitudes to depression, and in particular the possible association with employee mental health [47]. From our findings and the gender difference found in model 5, we found that the direction of the gender difference remains unaltered even after the inclusion of all the covariates.

Lastly, we found that the most unfavourable result for men was the report of being less comfortable dealing with staff members who have depression. This might be explained by the gendered expectations for masculine-stereotyped patterns of on-the-job behaviour [23] and a lower degree of open-mindedness to persons with mental illness among men, which was also found in other research [27].

From the results of the crude differences within each item between women and men and by looking at the age groups and distribution of women and men among the staff, we can confirm that gendered attitudes comply with the configurations of gender practice “masculinity” and “femininity” determined by society but also with the relationship pattern called gender regime constituted by the workplace [31]. The gender effect is only partly explained by the covariates included, but the direction did not change, confirming the gendered attitudes.

Considering that Sweden is a country where there is a low level of support for the male breadwinner model and a notable emphasis on gender equality [20], even though women are still outnumbered as managers [21], gender differences in terms of negative attitudes towards employees with depression might be less marked than elsewhere, as shown in Table 5 [48].

**Table 5:** Stigma is still widespread but people know mental illness can be treated

	People with mental health problems constitute a danger to others (2005)^a^				It is difficult to talk to someone with a significant mental health problem (2010)				People with mental health problems never recover (2006)			
	Severe	Moderate	None	Total	Severe	Moderate	None	Total	Severe	Moderate	None	Total
Austria	15.8	23.6	34.6	32.4	34.1	29.8	25.8	27.0	25.8	19.8	24.5	23.9
Belgium	35.3	26.7	30.9	30.9	37.0	29.5	21.7	23.8	24.0	28.1	18.1	19.1
Denmark	33.3	38.4	46.3	44.5	30.6	22.0	20.5	21.3	16.7	15.3	17.0	16.8
Netherlands	18.9	20.0	26.6	25.4	13.0	25.0	15.8	17.0	13.5	12.9	13.4	13.3
Sweden	55.3	51.0	56.7	55.9	18.9	18.8	13.1	14.4	18.8	18.4	13.7	14.7
United Kingdom	36.1	30.2	43.3	41.7	23.9	17.6	21.3	20.8	19.5	11.1	16.1	15.8
Average (21)	32.5	31.7	39.7	38.5	26.2	23.8	19.7	20.7	19.7	17.6	17.1	17.3
Standard deviation	14.2	11.4	11.1	11.1	9.3	5.2	4.5	4.5	4.6	6.1	4.0	3.8

Proportion of people who totally agree or tend to agree to a number of attitudinal questions, according to the level of mental health of the respondent (severe/moderate/no mental disorder) amended from Ref. ([48] , p 33). The average refers to all 21 countries covered in the Eurobarometer. Source: OECD compilation based on Eurobarometer 2005 and 2010

^a^The figures for Sweden might have been influenced by the murder of the leading politician Anna Lind in 2003 by a person treated for psychiatric disease

### Methodological strengths and limitations

The strengths of our study include the use of a valid and reliable instrument (MSED) [16] and of a large sample of more than 2500 managers representing a considerable variety of sectors; the samples used were suitable for exploring relationships and represent the distribution of men and women as managers in Sweden [21] as well as the Swedish labour market. Moreover, our study had a cross-sectional design, which is an important method for assessing the prevalence of phenomena as well as preliminary associations [49]. The outcomes of this study could be generalizable to other countries, and the difference could be even more pronounced in other countries with a lower degree of gender equality. We included all the covariates in the final model, including the social context influence, which is seldom taken into consideration in stigma studies [18]. This study did not include questions regarding gender issues in the measurements apart from the self-assigned categorical question at the beginning and the question about the distribution of women and men among the staff. The response bias was reduced by the introduction of two reverse-phrased items. The gendered experience was described in a dichotomized way as comparisons between women and men, which is the common cultural point of reference. However, the gender regime has to be considered as temporary and with reference to the contemporary point in time and history. Nevertheless, some limitations are present in the study. Among the sample from the Citizen Panel (70% of the non-responders), more men and managers aged 50–65 years refrained from participation, and more managers with a college/university degree participated compared with the non-responders. A similar investigation was not possible for the HELIX sample. We do not assume a gender bias in participation since the organizations and managers were approached with an information letter that focussed solely on the main objective of the present project, which is mental health at work. Both samples cannot be considered random, and the sample size for the non-binary category was too small to be used effectively in our analyses. As a result of the cross-sectional design, it was not possible to define causal relationships. Because self-assessed measurements for attitudes were used, different types of bias need to be considered, including social desirability, selection bias and responses biased by mere underestimation of one’s own negative attitudes. Managers might have answered according to what is socially acceptable due to embarrassment or discomfort about revealing true attitudes. Data on employees’ ratings of their managers’ depression attitudes would have been an important complementary perspective.

Because of the complexity of how gender is defined in modern gender theory, as explained in the background section, it is important to specify that the variable “gender” was used as an indicator of gendered experiences.

### Research recommendations

This study needs to be replicated in other countries to further investigate the distribution of negative attitudes among women and men. Future studies should study the association between gender and attitudes in different type of managerial positions, in different sectors, in relation to managers’ own experience and, in particular, the effect of health education to increase managers’ mental health literacy. We expect that positive attitudes would help reduce sickness absence and maintain people at work, but it could be that positive attitudes might make employees stay longer at home and might take place where females are managers and where we observe higher levels of sickness absence. Studies are necessary on the effects of attitudes (negative or positive) on sickness absence.

### Practical recommendations

Actions should be taken to reduce mental health stigma in the workplace [50]. What this study adds is that training programmes should address both male and female attitudes, or have different training programmes for male and female managers. Nowadays, training might be biased towards the female, positive attitude towards mental illness, and male managers might not be inspired. Preferably, managers should be trained to engage positively with employees who have depression or any other CMD as a part of their general management training [50].

## Conclusion

In this study, male managers had more negative attitudes towards employees with depression compared with female managers. This study is the first to investigate the role of gender in managers’ attitudes towards employees with depression. Our findings showed that a gender difference is indeed present in negative attitudes irrespective of age, education, sector, managerial position, current workplace experience in management, lifetime experience in management experience, distribution of women and men among the staff and the presence of staff members at the current workplace who have had depression and/or anxiety disorders. Based on these findings, a gender specific approach is suggested for future interventions to change attitudes in managers.

## Supplementary Information

**Additional file 1 Appendix**. Crude and adjusted odds ratio (OR) with 95% confidence interval (CI) for low negative attitudes compared with high negative attitudes in Swedish managers (*N* = 2663 of which 1762 were men and 901 were women): results for the covariates of binary logistic regression analyses, 2018.

## Data Availability

The data used for this study are archived at the Laboratory of Opinion Research (LORE) at the University of Gothenburg and can be obtained by contacting LORE at info@lore.gu.se.

## Abbreviations

CMD: Common mental disorder
LORE: Laboratory of Opinion Research at the University of Gothenburg
MSED: Managerial stigma towards employee depression
