# Supplementary material for: Gender differences in managers’ attitudes towards employees with depression: a cross-sectional study in Sweden
Source: BMC Public Health. 2020 Nov 19;20:1744. doi: 10.1186/s12889-020-09848-2 (PMC7678207; doi:10.1186/s12889-020-09848-2)
Supplement: Supplementary file 1 — Additional file 1 Appendix. Crude and adjusted odds ratio (OR) with 95% confidence interval (CI) for low negative attitudes compared with high negative attitudes in Swedish managers (N = 2663 of which 1762 were men and 901 were women): results for the covariates of binary logistic regression analyses, 2018. [file 12889_2020_9848_MOESM1_ESM.docx]

Appendix. Crude and adjusted odds ratio (OR) with 95% confidence interval (CI) for low negative attitudes compared with high negative attitudes in Swedish managers (*N* = 2663 of which 1762 were men and 901 were women): results for the covariates of binary logistic regression analyses, 2018

|  | Number | Negative attitudes towards depression | | | | | | | |
| --- | --- | --- | --- | --- | --- | --- | --- | --- | --- |
|  |  | Unadjusted OR (95% CI) | Model 1: OR (95% CI)^a^ | | | Model 2: OR (95% CI)^b^ | Model 3: OR (95% CI)^c^ | Model 4: OR (95% CI)^d^ | Model 5: OR (95% CI)^e^ |
| Age |  |  |  | | |  |  |  |  |
| Younger than 50 years | 1312 |  |  | | |  |  |  | 1 |
| 50 years and older | 1351 |  |  | | |  |  |  | 0.82 (.65- 1.03) |
| Level of education |  |  |  | | |  |  |  |  |
| Upper secondary school or lower | 406 |  | 1 | | | 1 | 1 | 1 | 1 |
| Degree from college/university | 1709 |  | 0.55 (0.43–0.70) | | | 0.66 (0.51–0.86) | 0.68 (0.52–0.89) | 0.67 (0.52–0.87) | 0.66 (0.51–0.86) |
| Other post-secondary education | 548 |  | 0.57 (0.43–0.77) | | | 0.61 (0.45–0.83) | 0.61 (0.45–0.82) | 0.61 (0.45–0.83) | 0.61 (0.45–0.83) |
| Work sector |  |  |  | | |  |  |  |  |
| Governmental | 360 |  |  | | | 1 | 1 | 1 | 1 |
| Municipal | 461 |  |  | | | 0.58 (0.38–0.88) | 0.57 (0.38–0.86) | 0.57 (0.38–0.87) | 0.58 (0.38–0.87) |
| County council/regional | 128 |  |  | | | 0.57 (0.31–1.07) | 0.55 (0.30–1.03) | 0.55 (0.29–1.03) | 0.56 (0.30–1.05) |
| Private | 1539 |  |  | | | 1.33 (0.98–1.80) | 1.11 (0.81–1.52) | 1.08 (0.79–1.48) | 1.06 (0.77–1.45) |
| Non-profit organization/foundation | 175 |  |  | | | 0.66 (0.39–1.13) | 0.54 (0.31–0.93) | 0.55 (0.32–0.95) | 0.55 (0.32–0.95) |
| Distribution of women and men among the staff |  |  |  | | |  |  |  |  |
| Most are women | 938 |  |  | | | 1 | 1 | 1 | 1 |
| There are about as many women as men | 734 |  |  | | | 0.83 (0.63–1.09) | 0.81 (0.61–1.06) | 0.81 (0.61–1.07) | 0.81 (0.62–1.07) |
| Most are men | 991 |  |  | | | 1.09 (0.84–1.41) | 1.11 (0.85–1.44) | 1.08 (0.83–1.40) | 1.07 (0.83–1.40) |
| Current workplace experience in management |  |  | |  |  | |  |  |  |
| 5 years or less | 1508 |  | |  |  | | 1 | 1 | 1 |
| More than 5 years | 1155 |  | |  |  | | 1.31 (1.07–1.61) | 1.33 (1.08–1.62) | 1.33 (1.07–1.65) |
| Lifetime experience in management |  |  | |  |  | |  |  |  |
| 10 years or less | 1314 |  | |  |  | | 1 | 1 | 1 |
| More than 10 years | 1349 |  | |  |  | |  |  | 1.14 (0.89–1.46) |
| Managerial position |  |  | |  |  | |  |  |  |
| Senior manager (such as administration manager, managing director) | 610 |  | |  |  | | 1 | 1 | 1 |
| Middle management (manager of managers) | 447 |  | |  |  | | 0.57 (0.42–0.78) | 0.59 (0.43–0.80) | 0.59 (0.43–0.80) |
| Middle management/first-line manager | 863 |  | |  |  | | 0.54 (0.41–0.72) | 0.56 (0.42–0.74) | 0.56 (0.42–0.75) |
| Group leader/supervisor | 535 |  | |  |  | | 0.67 (0.50–0.91) | 0.69 (0.51–0.92) | 0.69 (0.51–0.94) |
| Expert/operations manager (such as personnel manager, finance manager) | 208 |  | |  |  | | 0.77 (0.53–1.14) | 0.78 (0.53–1.15) | 0.79 (0.54–1.17) |
| Presence of staff members at current workplace who have had depression and/or anxiety disorders |  |  | |  |  | |  |  |  |
| Yes, one or more staff members | 1710 |  | |  |  | |  | 1 | 1 |
| No, no staff member | 759 |  | |  |  | |  | 1.32 (1.06–1.63) | 1.33 (1.07–1.65) |
| Don't know | 194 |  | |  |  | |  | 1.50 (1.05–2.14) | 1.51 (1.05–2.15) |

^a^Adjusted for level of education (age removed).

^b^Adjusted for level of education, sector and distribution of women and men among the staff.

^c^Adjusted for level of education, sector, distribution of women and men among the staff, current workplace experience in management and managerial position (lifetime experience in management removed).

^d^Adjusted for level of education, sector, distribution of women and men among the staff, current workplace experience in management, managerial position and presence of staff members at current workplace who have had depression and/or anxiety disorders.

^e^Adjusted for age, level of education, sector, distribution of women and men among the staff, current workplace experience in management, lifetime experience in management, managerial position and presence of staff members at current workplace who have had depression and/or anxiety disorders.
